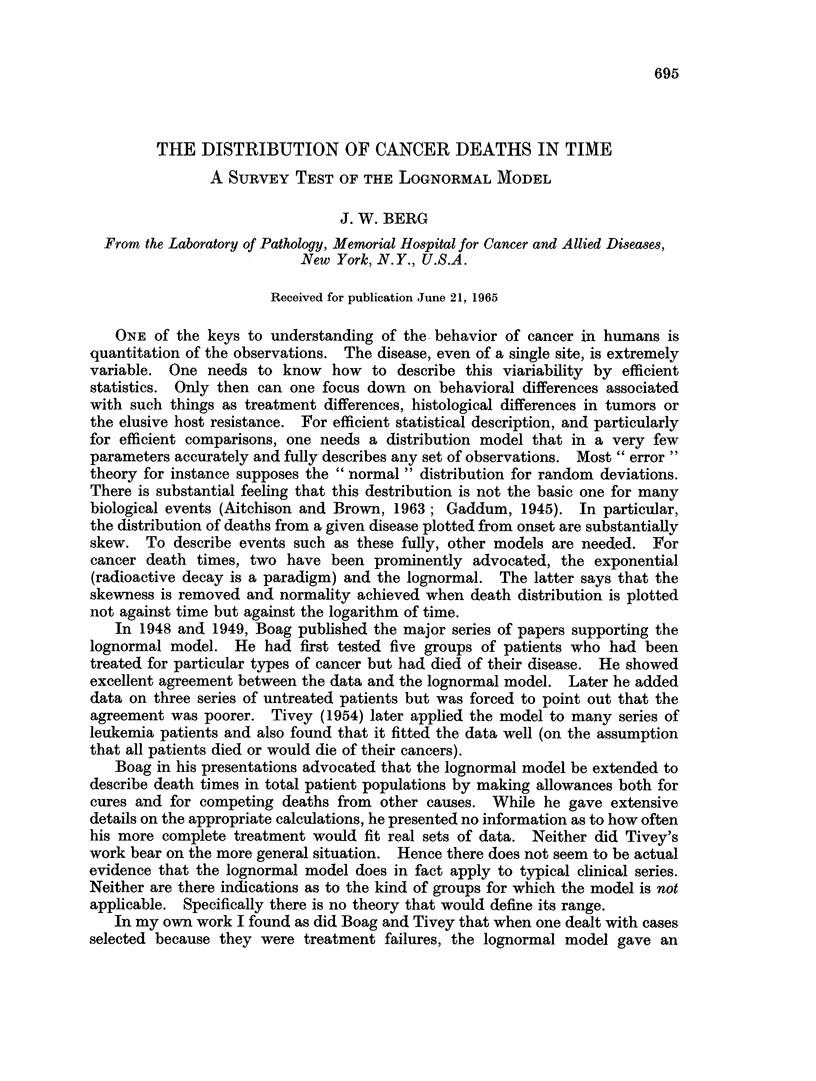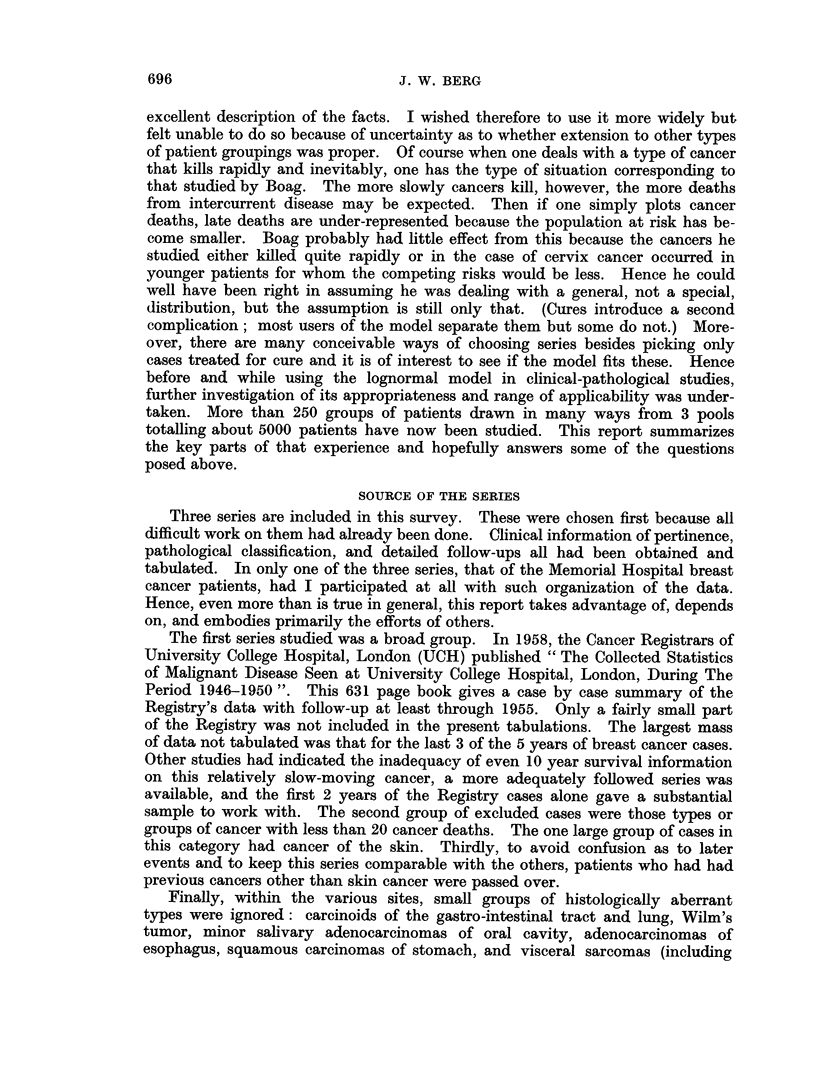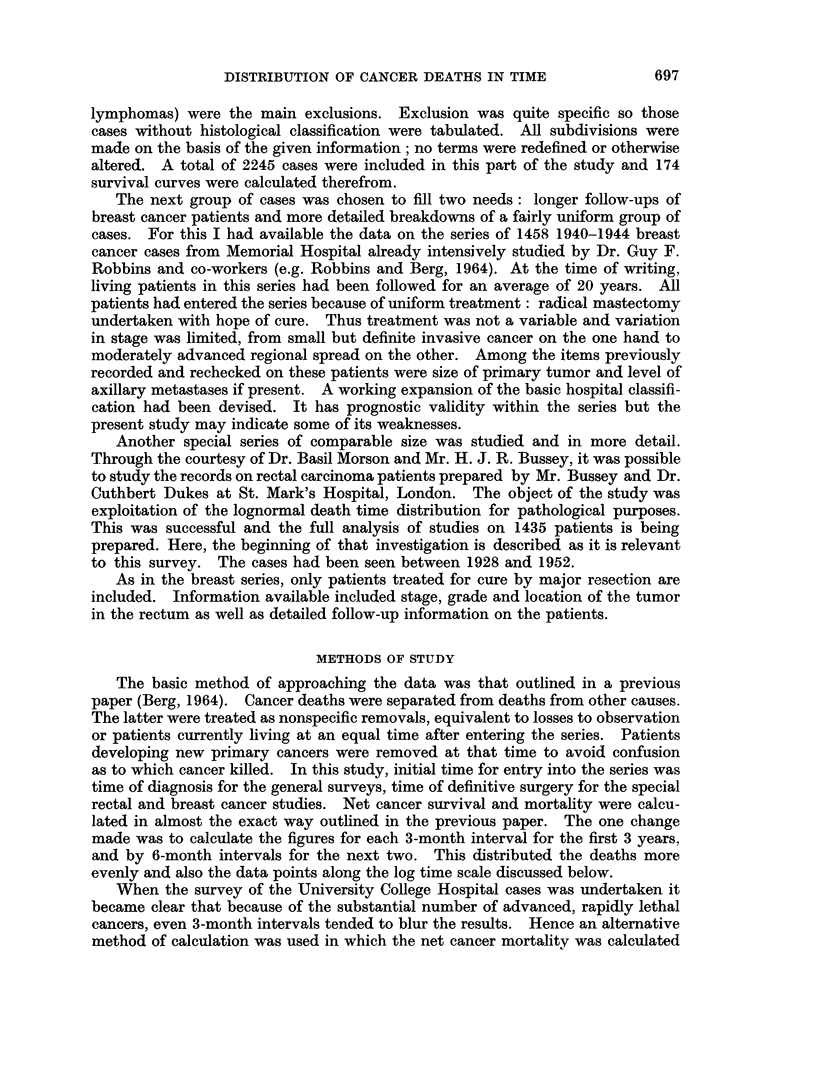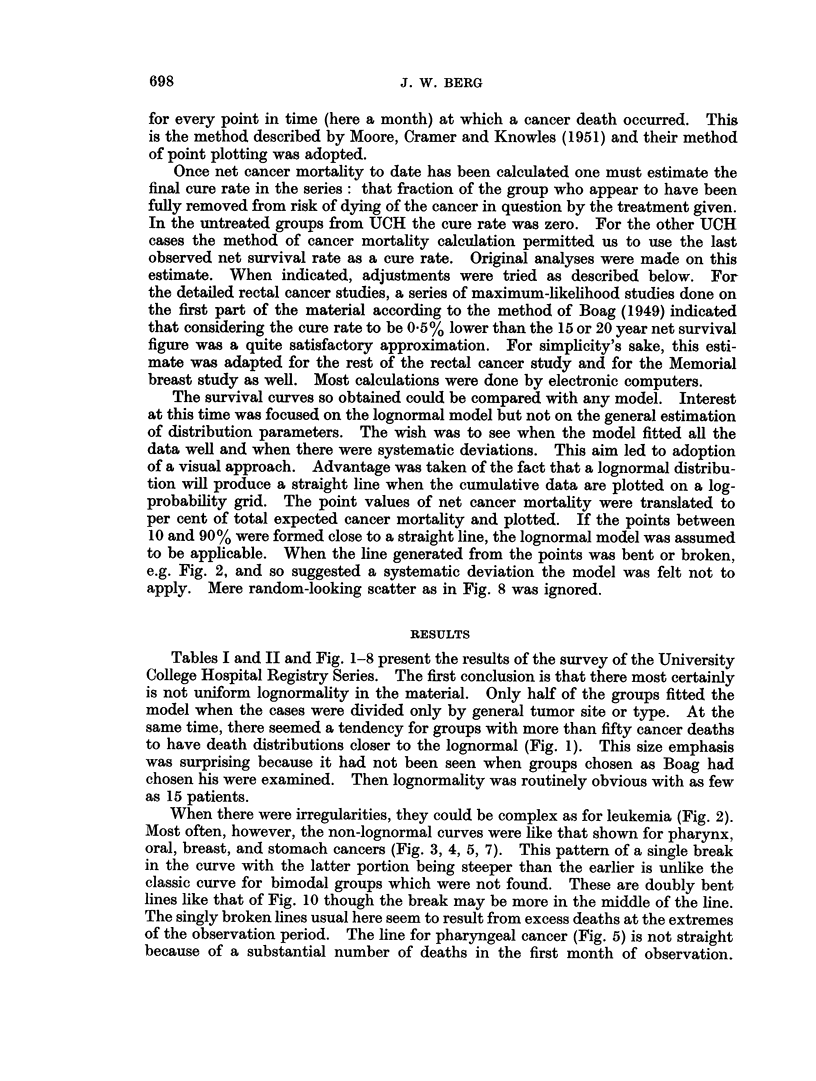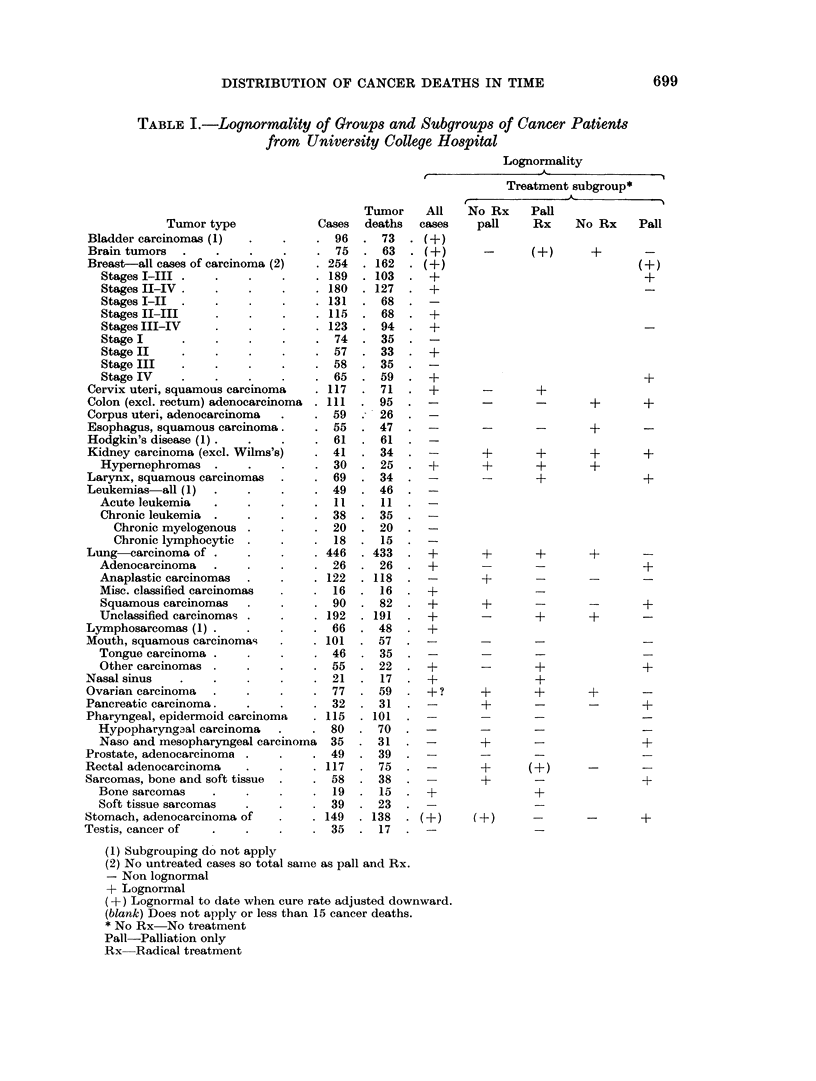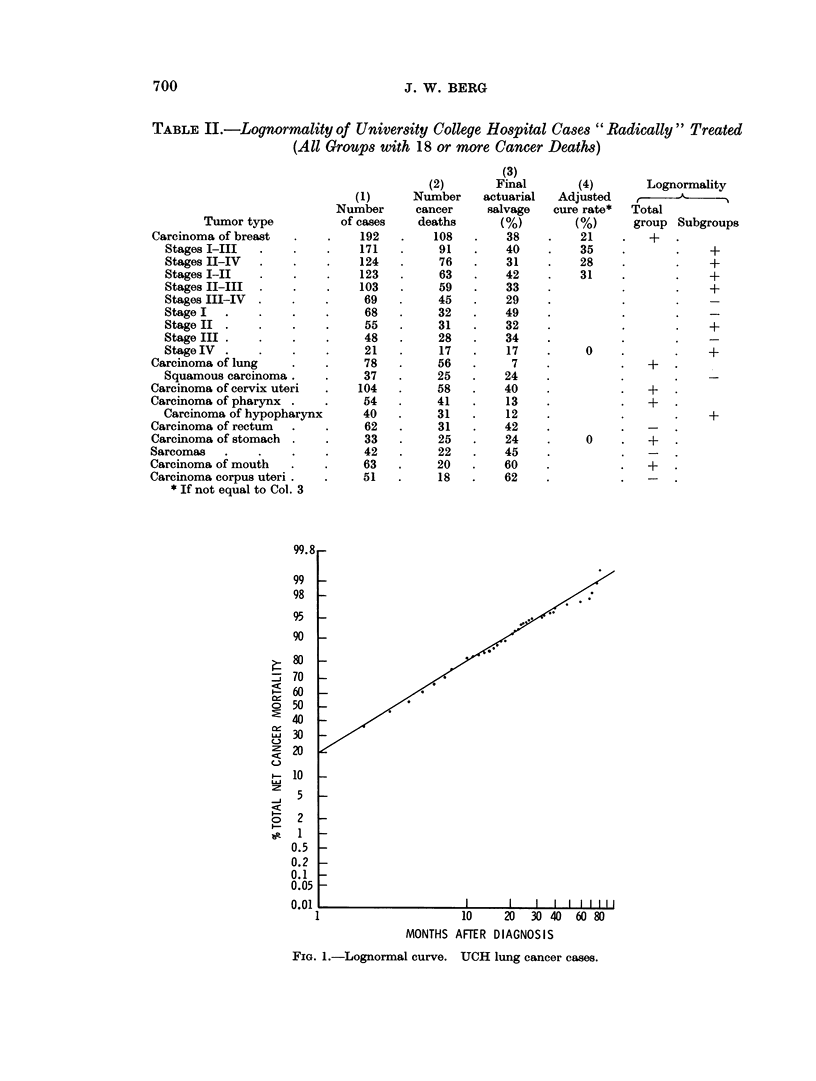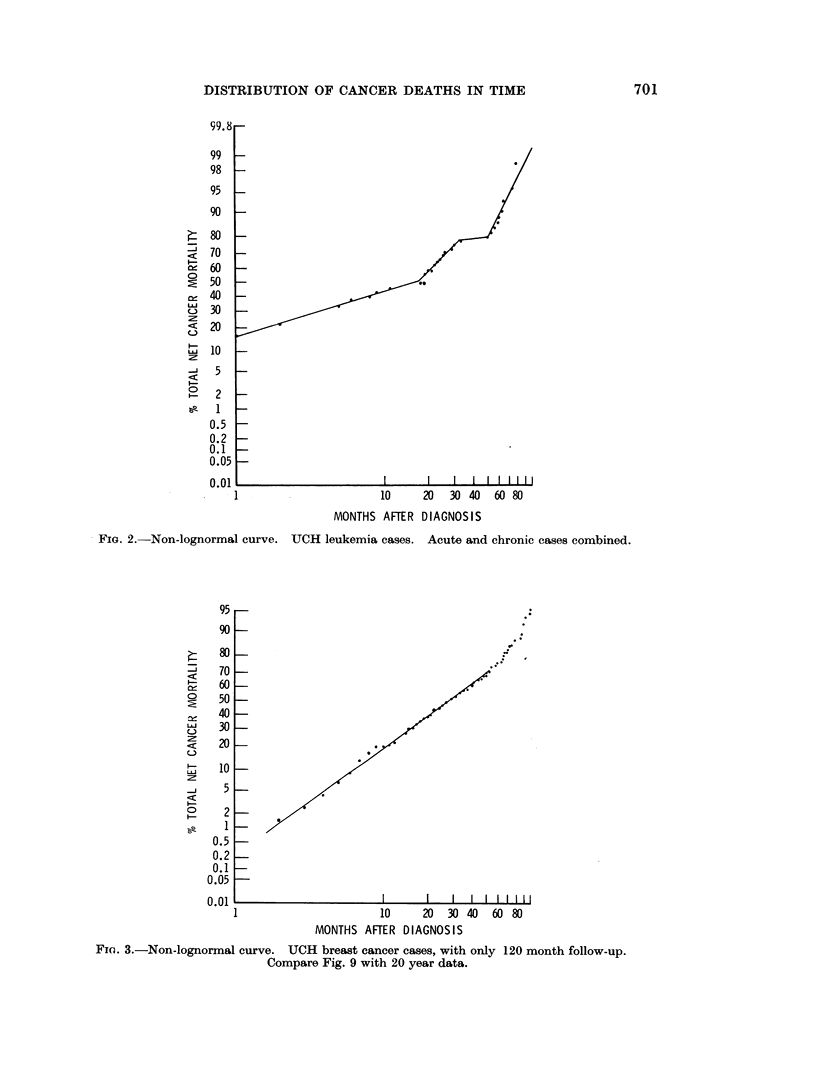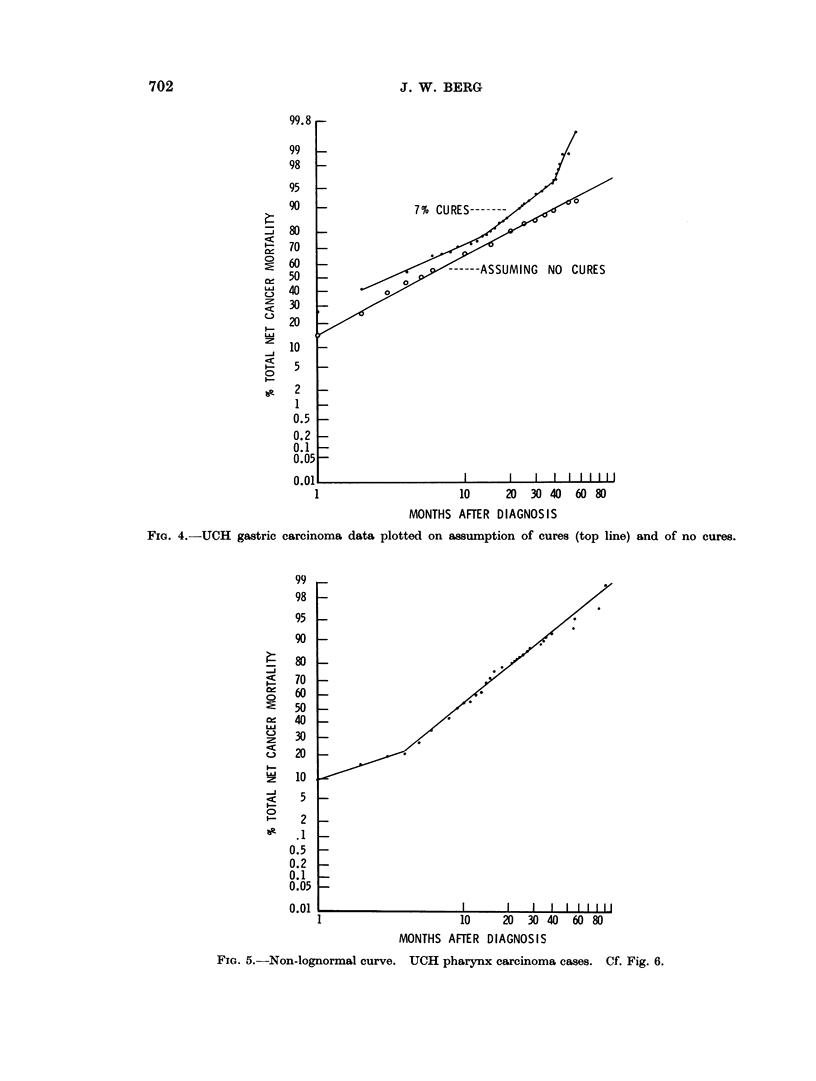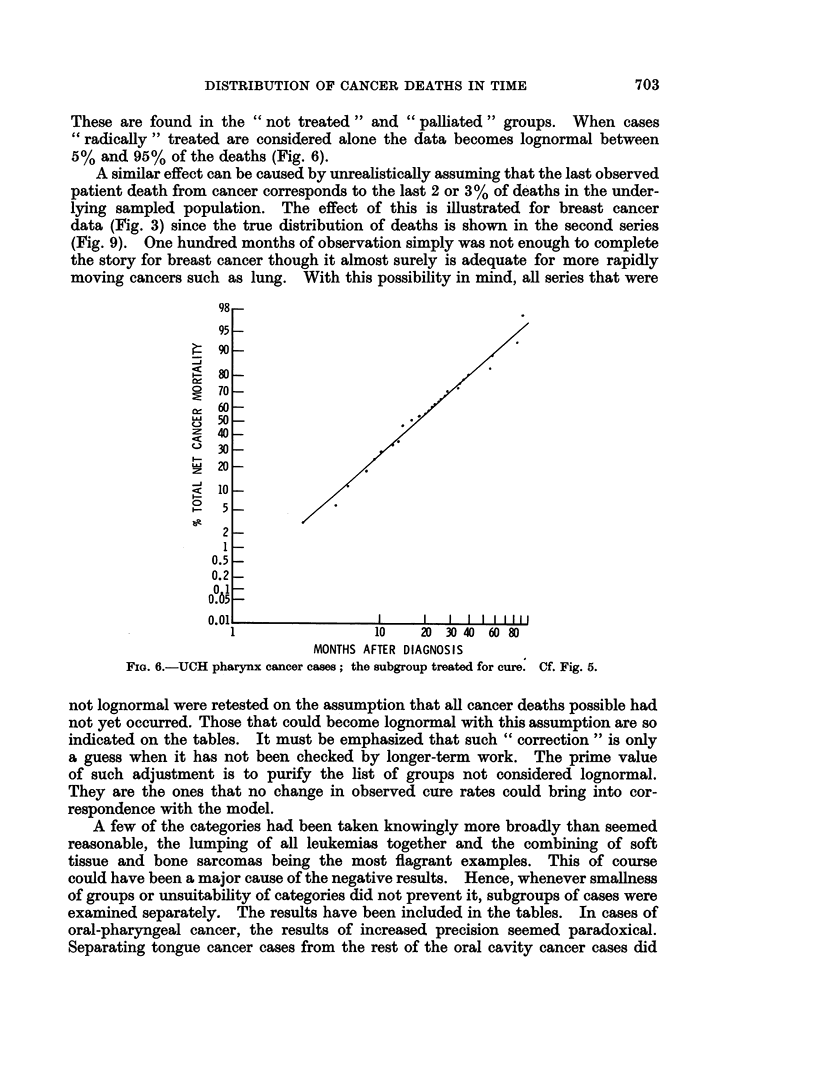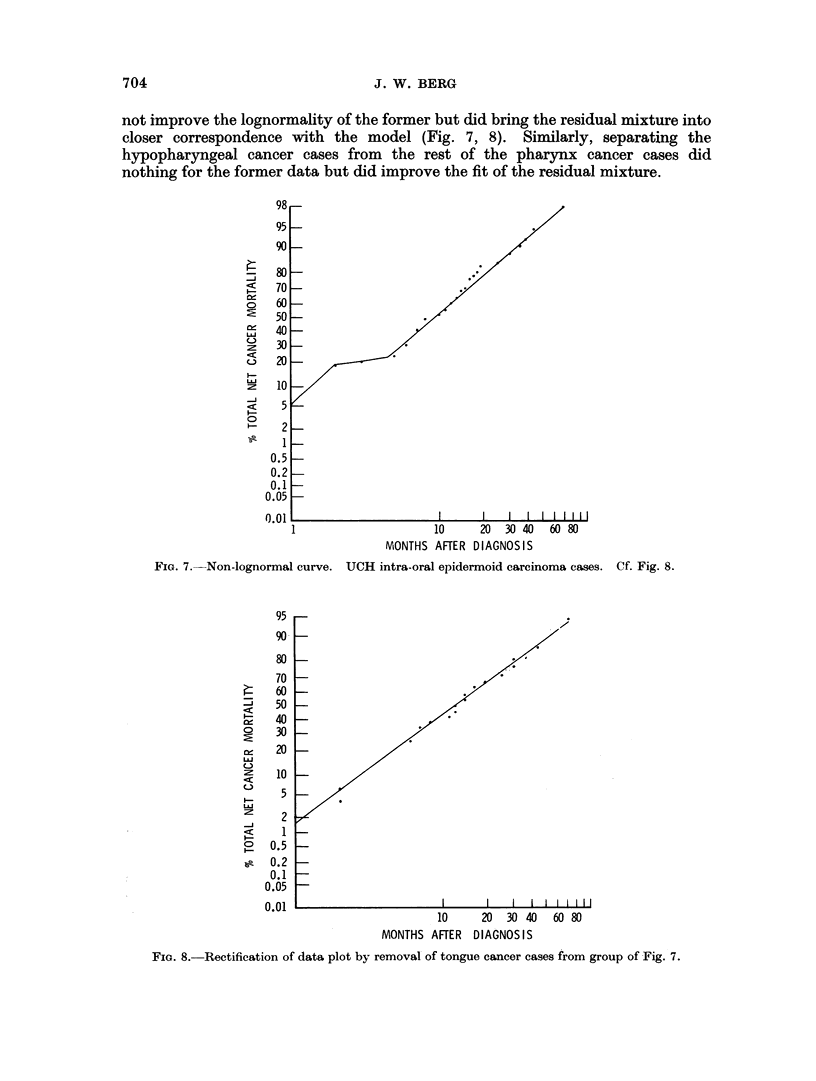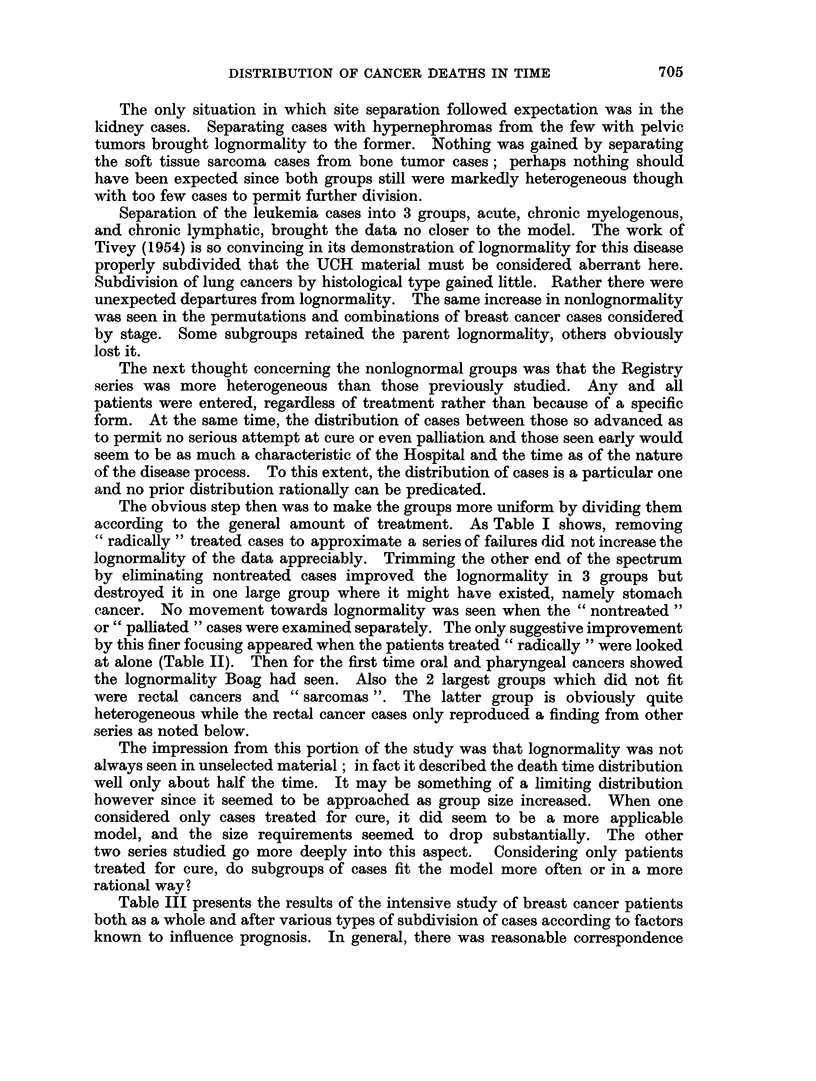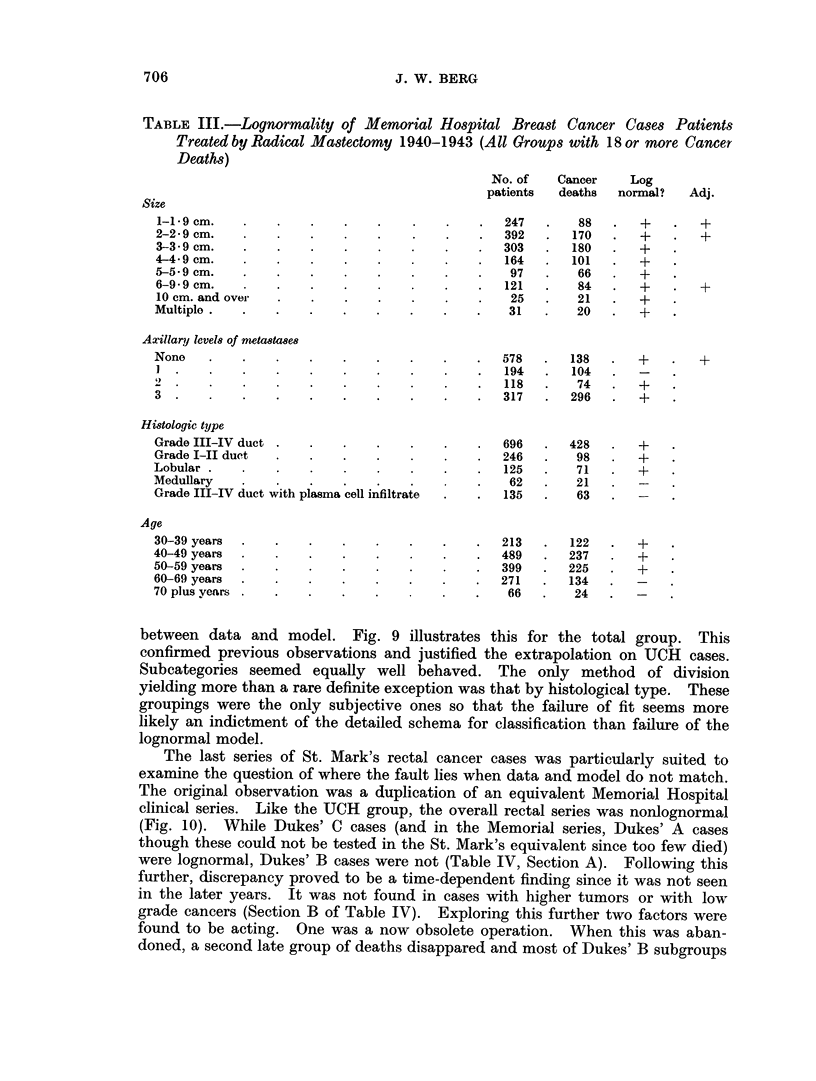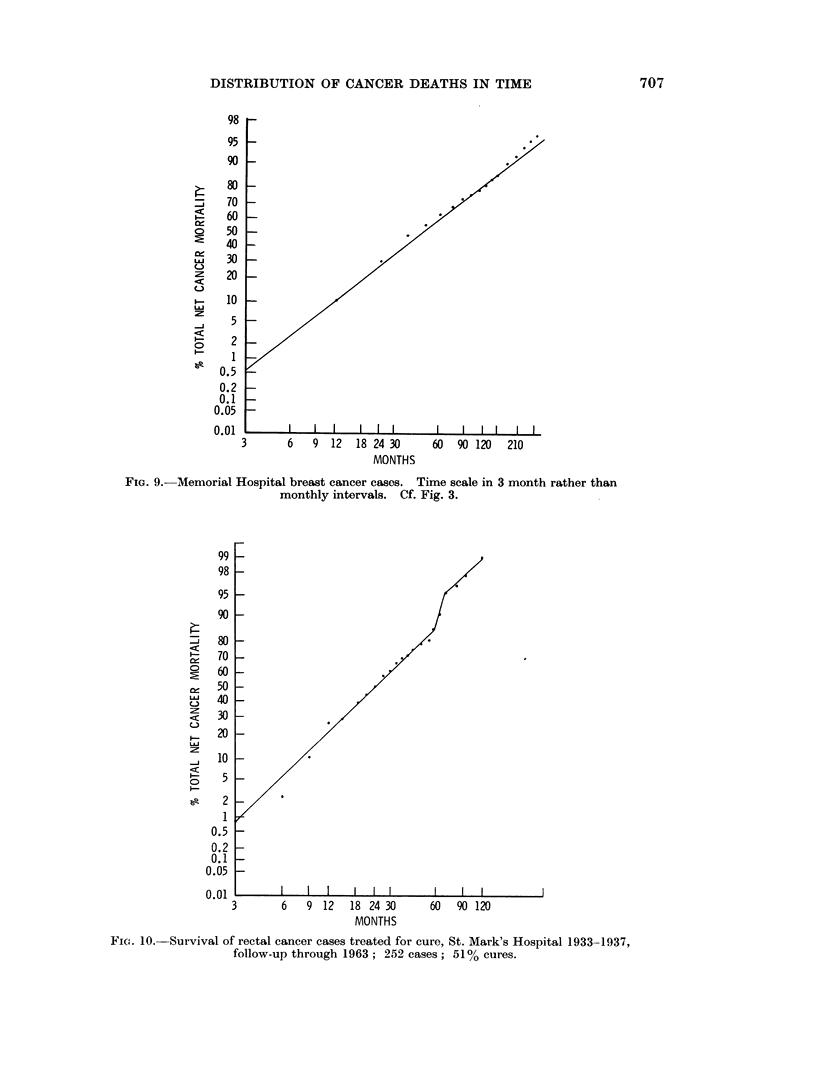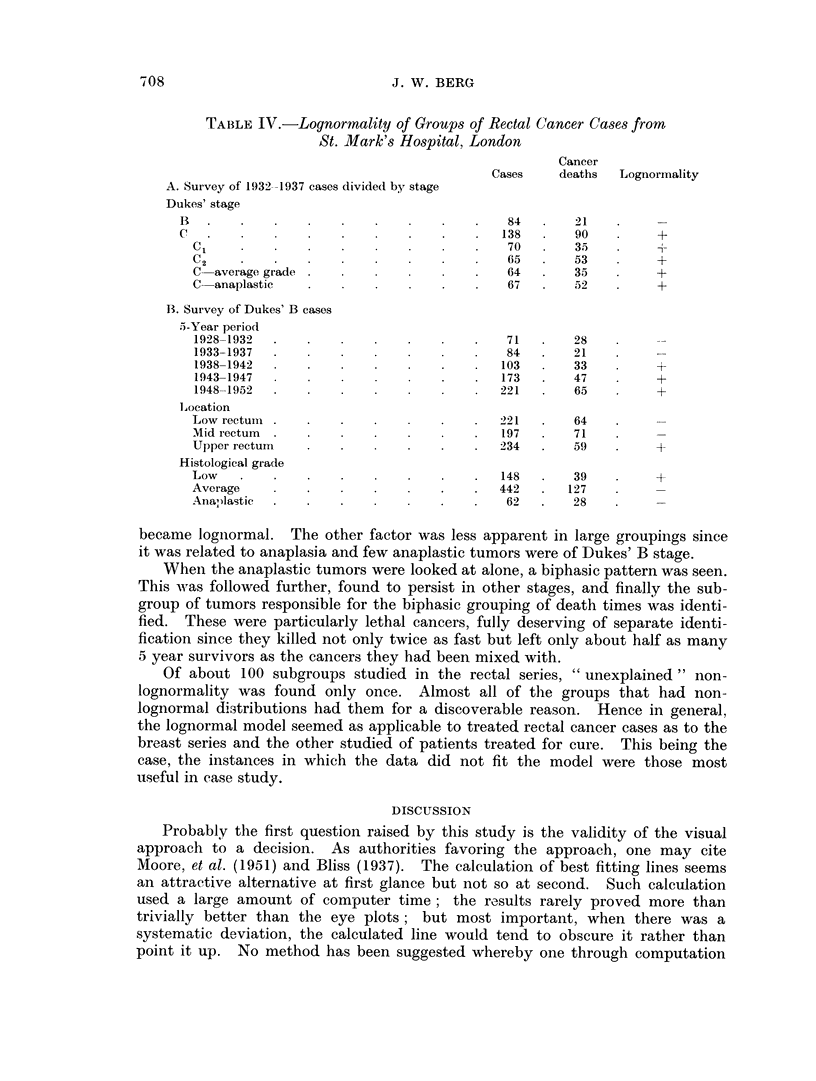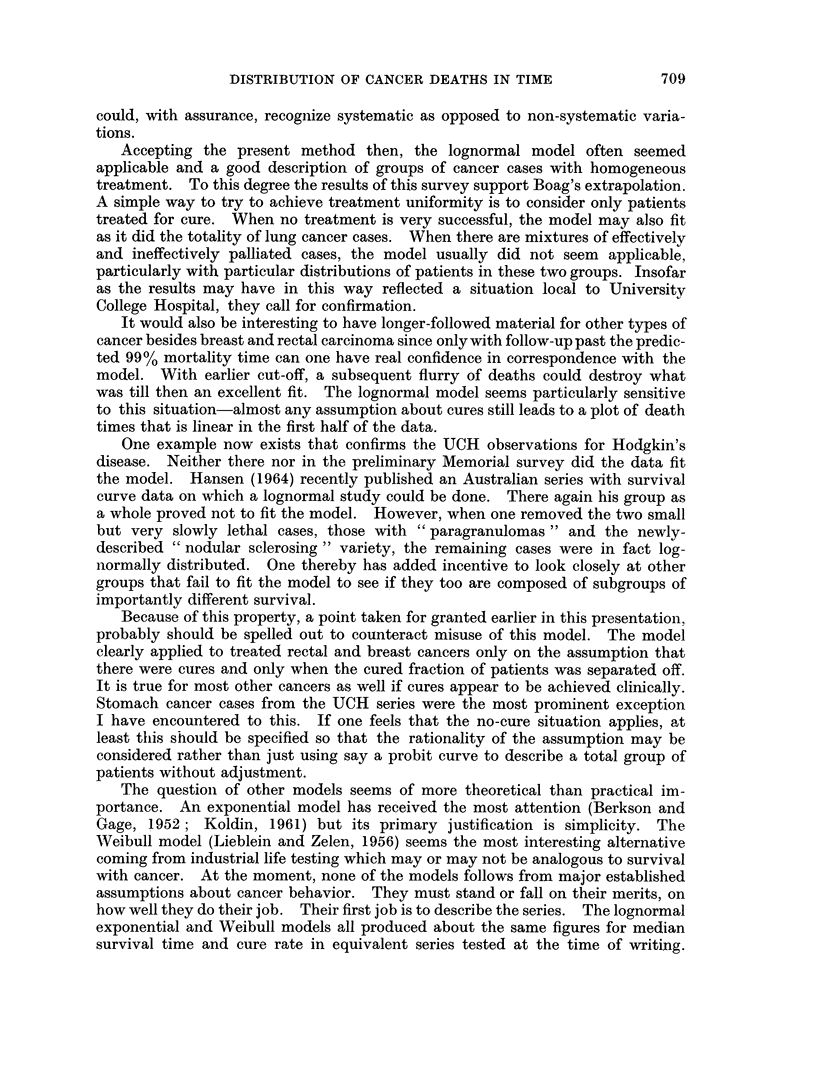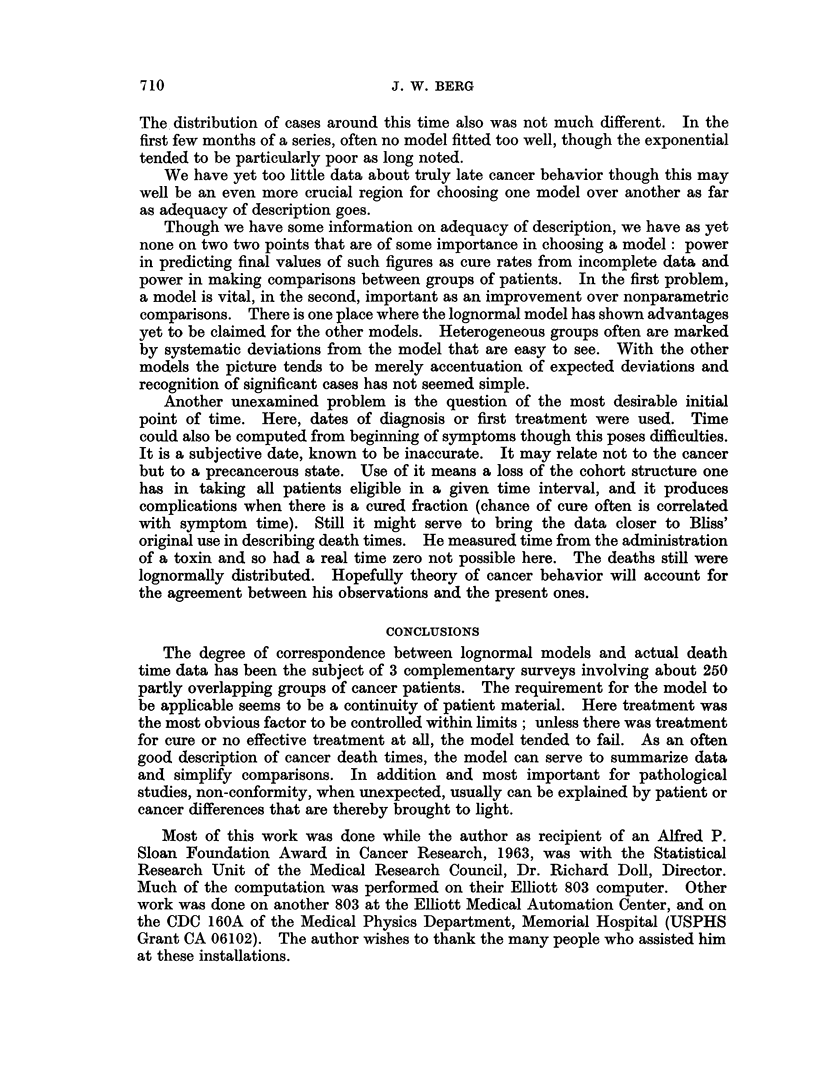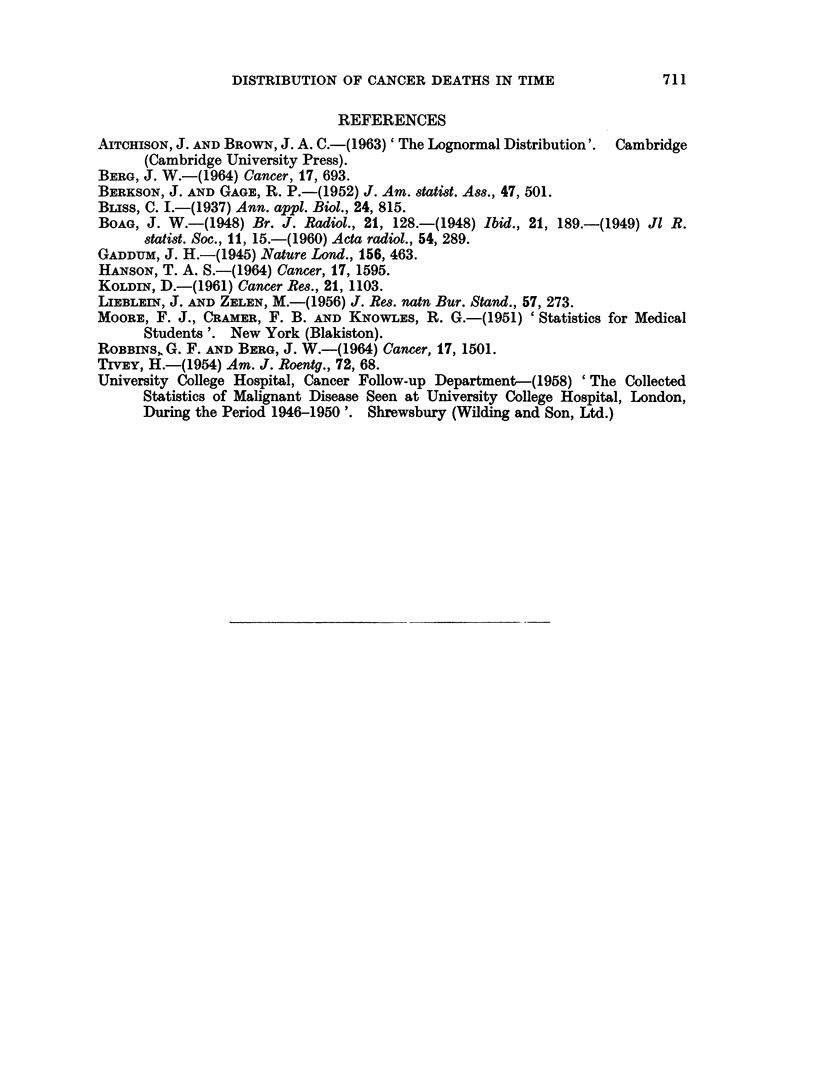# The distribution of cancer deaths in time. A survey test of the lognormal model.

**DOI:** 10.1038/bjc.1965.83

**Published:** 1965-12

**Authors:** J. W. Berg


### THE DISTRIBUTION OF CANCER DEATHS IN TIME

### A Survey Test of the Lognormal Model

J. W. BERG

*From the Laboratory of Pathology, Memorial Hospital for Cancer and Allied Diseases, New York, N.Y., U.S.A.*

Received for publication June 21, 1965

ONE of the keys to understanding of the behavior of cancer in humans is quantitation of the observations. The disease, even of a single site, is extremely variable. One needs to know how to describe this viariability by efficient statistics. Only then can one focus down on behavioral differences associated with such things as treatment differences, histological differences in tumors or the elusive host resistance. For efficient statistical description, and particularly for efficient comparisons, one needs a distribution model that in a very few parameters accurately and fully describes any set of observations. Most " error " theory for instance supposes the " normal " distribution for random deviations. There is substantial feeling that this destribution is not the basic one for many biological events (Aitchison and Brown, 1963 ; Gaddum, 1945). In particular, the distribution of deaths from a given disease plotted from onset are substantially skew. To describe events such as these fully, other models are needed. For cancer death times, two have been prominently advocated, the exponential (radioactive decay is a paradigm) and the lognormal. The latter says that the skewness is removed and normality achieved when death distribution is plotted not against time but against the logarithm of time.

In 1948 and 1949, Boag published the major series of papers supporting the lognormal model. He had first tested five groups of patients who had been treated for particular types of cancer but had died of their disease. He showed excellent agreement between the data and the lognormal model. Later he added data on three series of untreated patients but was forced to point out that the agreement was poorer. Tivey (1954) later applied the model to many series of leukemia patients and also found that it fitted the data well (on the assumption that all patients died or would die of their cancers).

Boag in his presentations advocated that the lognormal model be extended to describe death times in total patient populations by making allowances both for cures and for competing deaths from other causes. While he gave extensive details on the appropriate calculations, he presented no information as to how often his more complete treatment would fit real sets of data. Neither did Tivey's work bear on the more general situation. Hence there does not seem to be actual evidence that the lognormal model does in fact apply to typical clinical series. Neither are there indications as to the kind of groups for which the model is *not* applicable. Specifically there is no theory that would define its range.

In my own work I found as did Boag and Tivey that when one dealt with cases selected because they were treatment failures, the lognormal model gave an excellent description of the facts. I wished therefore to use it more widely but felt unable to do so because of uncertainty as to whether extension to other types of patient groupings was proper. Of course when one deals with a type of cancer that kills rapidly and inevitably, one has the type of situation corresponding to that studied by Boag. The more slowly cancers kill, however, the more deaths from intercurrent disease may be expected. Then if one simply plots cancer deaths, late deaths are under-represented because the population at risk has become smaller. Boag probably had little effect from this because the cancers he studied either killed quite rapidly or in the case of cervix cancer occurred in younger patients for whom the competing risks would be less. Hence he could well have been right in assuming he was dealing with a general, not a special, distribution, but the assumption is still only that. (Cures introduce a second complication ; most users of the model separate them but some do not.) Moreover, there are many conceivable ways of choosing series besides picking only cases treated for cure and it is of interest to see if the model fits these. Hence before and while using the lognormal model in clinical-pathological studies, further investigation of its appropriateness and range of applicability was undertaken. More than 250 groups of patients drawn in many ways from 3 pools totalling about 5000 patients have now been studied. This report summarizes the key parts of that experience and hopefully answers some of the questions posed above.

### SOURCE OF THE SERIES

Three series are included in this survey. These were chosen first because all difficult work on them had already been done. Clinical information of pertinence, pathological classification, and detailed follow-ups all had been obtained and tabulated. In only one of the three series, that of the Memorial Hospital breast cancer patients, had I participated at all with such organization of the data. Hence, even more than is true in general, this report takes advantage of, depends on, and embodies primarily the efforts of others.

The first series studied was a broad group. In 1958, the Cancer Registrars of University College Hospital, London (UCH) published " The Collected Statistics of Malignant Disease Seen at University College Hospital, London, During The Period 1946–1950 ". This 631 page book gives a case by case summary of the Registry's data with follow-up at least through 1955. Only a fairly small part of the Registry was not included in the present tabulations. The largest mass of data not tabulated was that for the last 3 of the 5 years of breast cancer cases. Other studies had indicated the inadequacy of even 10 year survival information on this relatively slow-moving cancer, a more adequately followed series was available, and the first 2 years of the Registry cases alone gave a substantial sample to work with. The second group of excluded cases were those types or groups of cancer with less than 20 cancer deaths. The one large group of cases in this category had cancer of the skin. Thirdly, to avoid confusion as to later events and to keep this series comparable with the others, patients who had had previous cancers other than skin cancer were passed over.

Finally, within the various sites, small groups of histologically aberrant types were ignored : carcinoids of the gastro-intestinal tract and lung, Wilm's tumor, minor salivary adenocarcinomas of oral cavity, adenocarcinomas of esophagus, squamous carcinomas of stomach, and visceral sarcomas (including lymphomas) were the main exclusions. Exclusion was quite specific so those cases without histological classification were tabulated. All subdivisions were made on the basis of the given information ; no terms were redefined or otherwise altered. A total of 2245 cases were included in this part of the study and 174 survival curves were calculated therefrom.

The next group of cases was chosen to fill two needs : longer follow-ups of breast cancer patients and more detailed breakdowns of a fairly uniform group of cases. For this I had available the data on the series of 1458 1940–1944 breast cancer cases from Memorial Hospital already intensively studied by Dr. Guy F. Robbins and co-workers (e.g. Robbins and Berg, 1964). At the time of writing, living patients in this series had been followed for an average of 20 years. All patients had entered the series because of uniform treatment : radical mastectomy undertaken with hope of cure. Thus treatment was not a variable and variation in stage was limited, from small but definite invasive cancer on the one hand to moderately advanced regional spread on the other. Among the items previously recorded and rechecked on these patients were size of primary tumor and level of axillary metastases if present. A working expansion of the basic hospital classification had been devised. It has prognostic validity within the series but the present study may indicate some of its weaknesses.

Another special series of comparable size was studied and in more detail. Through the courtesy of Dr. Basil Morson and Mr. H. J. R. Bussey, it was possible to study the records on rectal carcinoma patients prepared by Mr. Bussey and Dr. Cuthbert Dukes at St. Mark's Hospital, London. The object of the study was exploitation of the lognormal death time distribution for pathological purposes. This was successful and the full analysis of studies on 1435 patients is being prepared. Here, the beginning of that investigation is described as it is relevant to this survey. The cases had been seen between 1928 and 1952.

As in the breast series, only patients treated for cure by major resection are included. Information available included stage, grade and location of the tumor in the rectum as well as detailed follow-up information on the patients.

### METHODS OF STUDY

The basic method of approaching the data was that outlined in a previous paper (Berg, 1964). Cancer deaths were separated from deaths from other causes. The latter were treated as nonspecific removals, equivalent to losses to observation or patients currently living at an equal time after entering the series. Patients developing new primary cancers were removed at that time to avoid confusion as to which cancer killed. In this study, initial time for entry into the series was time of diagnosis for the general surveys, time of definitive surgery for the special rectal and breast cancer studies. Net cancer survival and mortality were calculated in almost the exact way outlined in the previous paper. The one change made was to calculate the figures for each 3-month interval for the first 3 years, and by 6-month intervals for the next two. This distributed the deaths more evenly and also the data points along the log time scale discussed below.

When the survey of the University College Hospital cases was undertaken it became clear that because of the substantial number of advanced, rapidly lethal cancers, even 3-month intervals tended to blur the results. Hence an alternative method of calculation was used in which the net cancer mortality was calculated for every point in time (here a month) at which a cancer death occurred. This is the method described by Moore, Cramer and Knowles (1951) and their method of point plotting was adopted.

Once net cancer mortality to date has been calculated one must estimate the final cure rate in the series : that fraction of the group who appear to have been fully removed from risk of dying of the cancer in question by the treatment given. In the untreated groups from UCH the cure rate was zero. For the other UCH cases the method of cancer mortality calculation permitted us to use the last observed net survival rate as a cure rate. Original analyses were made on this estimate. When indicated, adjustments were tried as described below. For the detailed rectal cancer studies, a series of maximum-likelihood studies done on the first part of the material according to the method of Boag (1949) indicated that considering the cure rate to be 0·5% lower than the 15 or 20 year net survival figure was a quite satisfactory approximation. For simplicity's sake, this estimate was adapted for the rest of the rectal cancer study and for the Memorial breast study as well. Most calculations were done by electronic computers.

The survival curves so obtained could be compared with any model. Interest at this time was focused on the lognormal model but not on the general estimation of distribution parameters. The wish was to see when the model fitted all the data well and when there were systematic deviations. This aim led to adoption of a visual approach. Advantage was taken of the fact that a lognormal distribution will produce a straight line when the cumulative data are plotted on a log-probability grid. The point values of net cancer mortality were translated to per cent of total expected cancer mortality and plotted. If the points between 10 and 90% were formed close to a straight line, the lognormal model was assumed to be applicable. When the line generated from the points was bent or broken, e.g. Fig. 2, and so suggested a systematic deviation the model was felt not to apply. Mere random-looking scatter as in Fig. 8 was ignored.

### RESULTS

Tables I and II and Fig. 1–8 present the results of the survey of the University College Hospital Registry Series. The first conclusion is that there most certainly is not uniform lognormality in the material. Only half of the groups fitted the model when the cases were divided only by general tumor site or type. At the same time, there seemed a tendency for groups with more than fifty cancer deaths to have death distributions closer to the lognormal (Fig. 1). This size emphasis was surprising because it had not been seen when groups chosen as Boag had chosen his were examined. Then lognormality was routinely obvious with as few as 15 patients.

When there were irregularities, they could be complex as for leukemia (Fig. 2). Most often, however, the non-lognormal curves were like that shown for pharynx, oral, breast, and stomach cancers (Fig. 3, 4, 5, 7). This pattern of a single break in the curve with the latter portion being steeper than the earlier is unlike the classic curve for bimodal groups which were not found. These are doubly bent lines like that of Fig. 10 though the break may be more in the middle of the line. The singly broken lines usual here seem to result from excess deaths at the extremes of the observation period. The line for pharyngeal cancer (Fig. 5) is not straight because of a substantial number of deaths in the first month of observation.

TABLE I.—*Lognormality of Groups and Subgroups of Cancer Patients from University College Hospital*

| | | | Lognormality | | | | |
|---|---|---|---|---|---|---|---|
| | | | | Treatment subgroup* | | | |
| Tumor type | Cases | Tumor deaths | All cases | No Rx pall | Pall Rx | No Rx | Pall |
| Bladder carcinomas (1) | 96 | 73 | (+) | | | | |
| Brain tumors | 75 | 63 | (+) | – | (+) | + | – |
| Breast—all cases of carcinoma (2) | 254 | 162 | (+) | | | | (+) |
| Stages I–III | 189 | 103 | + | | | | + |
| Stages II–IV | 180 | 127 | + | | | | – |
| Stages I–II | 131 | 68 | – | | | | |
| Stages II–III | 115 | 68 | + | | | | |
| Stages III–IV | 123 | 94 | + | | | | – |
| Stage I | 74 | 35 | – | | | | |
| Stage II | 57 | 33 | + | | | | |
| Stage III | 58 | 35 | – | | | | |
| Stage IV | 65 | 59 | + | | | | + |
| Cervix uteri, squamous carcinoma | 117 | 71 | + | – | + | | |
| Colon (excl. rectum) adenocarcinoma | 111 | 95 | – | – | – | + | + |
| Corpus uteri, adenocarcinoma | 59 | 26 | – | | | | |
| Esophagus, squamous carcinoma | 55 | 47 | – | – | – | + | – |
| Hodgkin's disease (1) | 61 | 61 | – | | | | |
| Kidney carcinoma (excl. Wilms's) | 41 | 34 | – | + | + | + | + |
| Hypernephromas | 30 | 25 | + | + | + | + | |
| Larynx, squamous carcinomas | 69 | 34 | – | – | + | | + |
| Leukemias—all (1) | 49 | 46 | – | | | | |
| Acute leukemia | 11 | 11 | – | | | | |
| Chronic leukemia | 38 | 35 | – | | | | |
| Chronic myelogenous | 20 | 20 | – | | | | |
| Chronic lymphocytic | 18 | 15 | – | | | | |
| Lung—carcinoma of | 446 | 433 | + | + | + | + | – |
| Adenocarcinoma | 26 | 26 | + | – | – | | + |
| Anaplastic carcinomas | 122 | 118 | – | + | – | – | – |
| Misc. classified carcinomas | 16 | 16 | + | | – | | |
| Squamous carcinomas | 90 | 82 | + | + | – | – | + |
| Unclassified carcinomas | 192 | 191 | + | – | + | + | – |
| Lymphosarcomas (1) | 66 | 48 | + | | | | |
| Mouth, squamous carcinomas | 101 | 57 | – | – | – | | – |
| Tongue carcinoma | 46 | 35 | – | – | – | | – |
| Other carcinomas | 55 | 22 | + | – | + | | + |
| Nasal sinus | 21 | 17 | + | | + | | |
| Ovarian carcinoma | 77 | 59 | +? | + | + | + | – |
| Pancreatic carcinoma | 32 | 31 | – | + | – | – | + |
| Pharyngeal, epidermoid carcinoma | 115 | 101 | – | – | – | | – |
| Hypopharyngeal carcinoma | 80 | 70 | – | – | – | | – |
| Naso and mesopharyngeal carcinoma | 35 | 31 | – | + | – | | + |
| Prostate, adenocarcinoma | 49 | 39 | – | – | – | | – |
| Rectal adenocarcinoma | 117 | 75 | – | + | (+) | – | – |
| Sarcomas, bone and soft tissue | 58 | 38 | – | + | – | | + |
| Bone sarcomas | 19 | 15 | + | | + | | |
| Soft tissue sarcomas | 39 | 23 | – | | – | | |
| Stomach, adenocarcinoma of | 149 | 138 | (+) | (+) | – | – | + |
| Testis, cancer of | 35 | 17 | – | | – | | |

(1) Subgrouping do not apply
(2) No untreated cases so total same as pall and Rx.
– Non lognormal
+ Lognormal
(+) Lognormal to date when cure rate adjusted downward.
(*blank*) Does not apply or less than 15 cancer deaths.
* No Rx—No treatment
Pall—Palliation only
Rx—Radical treatment

TABLE II.—*Lognormality of University College Hospital Cases "Radically" Treated (All Groups with* 18 *or more Cancer Deaths)*

| Tumor type | (1) Number of cases | (2) Number cancer deaths | (3) Final actuarial salvage (%) | (4) Adjusted cure rate* (%) | Lognormality: Total group | Lognormality: Subgroups |
|---|---|---|---|---|---|---|
| Carcinoma of breast | 192 | 108 | 38 | 21 | + | |
| Stages I–III | 171 | 91 | 40 | 35 | | + |
| Stages II–IV | 124 | 76 | 31 | 28 | | + |
| Stages I–II | 123 | 63 | 42 | 31 | | + |
| Stages II–III | 103 | 59 | 33 | | | + |
| Stages III–IV | 69 | 45 | 29 | | | − |
| Stage I | 68 | 32 | 49 | | | − |
| Stage II | 55 | 31 | 32 | | | + |
| Stage III | 48 | 28 | 34 | | | − |
| Stage IV | 21 | 17 | 17 | 0 | | + |
| Carcinoma of lung | 78 | 56 | 7 | | + | |
| Squamous carcinoma | 37 | 25 | 24 | | | − |
| Carcinoma of cervix uteri | 104 | 58 | 40 | | + | |
| Carcinoma of pharynx | 54 | 41 | 13 | | + | |
| Carcinoma of hypopharynx | 40 | 31 | 12 | | | + |
| Carcinoma of rectum | 62 | 31 | 42 | | − | |
| Carcinoma of stomach | 33 | 25 | 24 | 0 | + | |
| Sarcomas | 42 | 22 | 45 | | − | |
| Carcinoma of mouth | 63 | 20 | 60 | | + | |
| Carcinoma corpus uteri | 51 | 18 | 62 | | − | |

* If not equal to Col. 3

FIG. 1.—Lognormal curve. UCH lung cancer cases.

FIG. 2.—Non-lognormal curve. UCH leukemia cases. Acute and chronic cases combined.

FIG. 3.—Non-lognormal curve. UCH breast cancer cases, with only 120 month follow-up. Compare Fig. 9 with 20 year data.

FIG. 4.—UCH gastric carcinoma data plotted on assumption of cures (top line) and of no cures.

FIG. 5.—Non-lognormal curve. UCH pharynx carcinoma cases. Cf. Fig. 6.

These are found in the " not treated " and " palliated " groups. When cases " radically " treated are considered alone the data becomes lognormal between 5% and 95% of the deaths (Fig. 6).

A similar effect can be caused by unrealistically assuming that the last observed patient death from cancer corresponds to the last 2 or 3% of deaths in the underlying sampled population. The effect of this is illustrated for breast cancer data (Fig. 3) since the true distribution of deaths is shown in the second series (Fig. 9). One hundred months of observation simply was not enough to complete the story for breast cancer though it almost surely is adequate for more rapidly moving cancers such as lung. With this possibility in mind, all series that were

FIG. 6.—UCH pharynx cancer cases ; the subgroup treated for cure. Cf. Fig. 5.

not lognormal were retested on the assumption that all cancer deaths possible had not yet occurred. Those that could become lognormal with this assumption are so indicated on the tables. It must be emphasized that such " correction " is only a guess when it has not been checked by longer-term work. The prime value of such adjustment is to purify the list of groups not considered lognormal. They are the ones that no change in observed cure rates could bring into correspondence with the model.

A few of the categories had been taken knowingly more broadly than seemed reasonable, the lumping of all leukemias together and the combining of soft tissue and bone sarcomas being the most flagrant examples. This of course could have been a major cause of the negative results. Hence, whenever smallness of groups or unsuitability of categories did not prevent it, subgroups of cases were examined separately. The results have been included in the tables. In cases of oral-pharyngeal cancer, the results of increased precision seemed paradoxical. Separating tongue cancer cases from the rest of the oral cavity cancer cases did not improve the lognormality of the former but did bring the residual mixture into closer correspondence with the model (Fig. 7, 8). Similarly, separating the hypopharyngeal cancer cases from the rest of the pharynx cancer cases did nothing for the former data but did improve the fit of the residual mixture.

FIG. 7.—Non-lognormal curve. UCH intra-oral epidermoid carcinoma cases. Cf. Fig. 8.

FIG. 8.—Rectification of data plot by removal of tongue cancer cases from group of Fig. 7.

The only situation in which site separation followed expectation was in the kidney cases. Separating cases with hypernephromas from the few with pelvic tumors brought lognormality to the former. Nothing was gained by separating the soft tissue sarcoma cases from bone tumor cases ; perhaps nothing should have been expected since both groups still were markedly heterogeneous though with too few cases to permit further division.

Separation of the leukemia cases into 3 groups, acute, chronic myelogenous, and chronic lymphatic, brought the data no closer to the model. The work of Tivey (1954) is so convincing in its demonstration of lognormality for this disease properly subdivided that the UCH material must be considered aberrant here. Subdivision of lung cancers by histological type gained little. Rather there were unexpected departures from lognormality. The same increase in nonlognormality was seen in the permutations and combinations of breast cancer cases considered by stage. Some subgroups retained the parent lognormality, others obviously lost it.

The next thought concerning the nonlognormal groups was that the Registry series was more heterogeneous than those previously studied. Any and all patients were entered, regardless of treatment rather than because of a specific form. At the same time, the distribution of cases between those so advanced as to permit no serious attempt at cure or even palliation and those seen early would seem to be as much a characteristic of the Hospital and the time as of the nature of the disease process. To this extent, the distribution of cases is a particular one and no prior distribution rationally can be predicated.

The obvious step then was to make the groups more uniform by dividing them according to the general amount of treatment. As Table I shows, removing " radically " treated cases to approximate a series of failures did not increase the lognormality of the data appreciably. Trimming the other end of the spectrum by eliminating nontreated cases improved the lognormality in 3 groups but destroyed it in one large group where it might have existed, namely stomach cancer. No movement towards lognormality was seen when the " nontreated " or " palliated " cases were examined separately. The only suggestive improvement by this finer focusing appeared when the patients treated " radically " were looked at alone (Table II). Then for the first time oral and pharyngeal cancers showed the lognormality Boag had seen. Also the 2 largest groups which did not fit were rectal cancers and " sarcomas ". The latter group is obviously quite heterogeneous while the rectal cancer cases only reproduced a finding from other series as noted below.

The impression from this portion of the study was that lognormality was not always seen in unselected material ; in fact it described the death time distribution well only about half the time. It may be something of a limiting distribution however since it seemed to be approached as group size increased. When one considered only cases treated for cure, it did seem to be a more applicable model, and the size requirements seemed to drop substantially. The other two series studied go more deeply into this aspect. Considering only patients treated for cure, do subgroups of cases fit the model more often or in a more rational way?

Table III presents the results of the intensive study of breast cancer patients both as a whole and after various types of subdivision of cases according to factors known to influence prognosis. In general, there was reasonable correspondence

TABLE III.—*Lognormality of Memorial Hospital Breast Cancer Cases Patients Treated by Radical Mastectomy* 1940–1943 (*All Groups with* 18 *or more Cancer Deaths*)

| | No. of patients | Cancer deaths | Log normal? | Adj. |
|---|---|---|---|---|
| *Size* | | | | |
| 1–1·9 cm. | 247 | 88 | + | + |
| 2–2·9 cm. | 392 | 170 | + | + |
| 3–3·9 cm. | 303 | 180 | + | |
| 4–4·9 cm. | 164 | 101 | + | |
| 5–5·9 cm. | 97 | 66 | + | |
| 6–9·9 cm. | 121 | 84 | + | + |
| 10 cm. and over | 25 | 21 | + | |
| Multiple | 31 | 20 | + | |
| *Axillary levels of metastases* | | | | |
| None | 578 | 138 | + | + |
| 1 | 194 | 104 | — | |
| 2 | 118 | 74 | + | |
| 3 | 317 | 296 | + | |
| *Histologic type* | | | | |
| Grade III–IV duct | 696 | 428 | + | |
| Grade I–II duct | 246 | 98 | + | |
| Lobular | 125 | 71 | + | |
| Medullary | 62 | 21 | — | |
| Grade III–IV duct with plasma cell infiltrate | 135 | 63 | — | |
| *Age* | | | | |
| 30–39 years | 213 | 122 | + | |
| 40–49 years | 489 | 237 | + | |
| 50–59 years | 399 | 225 | + | |
| 60–69 years | 271 | 134 | — | |
| 70 plus years | 66 | 24 | — | |

between data and model. Fig. 9 illustrates this for the total group. This confirmed previous observations and justified the extrapolation on UCH cases. Subcategories seemed equally well behaved. The only method of division yielding more than a rare definite exception was that by histological type. These groupings were the only subjective ones so that the failure of fit seems more likely an indictment of the detailed schema for classification than failure of the lognormal model.

The last series of St. Mark's rectal cancer cases was particularly suited to examine the question of where the fault lies when data and model do not match. The original observation was a duplication of an equivalent Memorial Hospital clinical series. Like the UCH group, the overall rectal series was nonlognormal (Fig. 10). While Dukes' C cases (and in the Memorial series, Dukes' A cases though these could not be tested in the St. Mark's equivalent since too few died) were lognormal, Dukes' B cases were not (Table IV, Section A). Following this further, discrepancy proved to be a time-dependent finding since it was not seen in the later years. It was not found in cases with higher tumors or with low grade cancers (Section B of Table IV). Exploring this further two factors were found to be acting. One was a now obsolete operation. When this was abandoned, a second late group of deaths disappeared and most of Dukes' B subgroups

FIG. 9.—Memorial Hospital breast cancer cases. Time scale in 3 month rather than monthly intervals. Cf. Fig. 3.

FIG. 10.—Survival of rectal cancer cases treated for cure, St. Mark's Hospital 1933–1937, follow-up through 1963 ; 252 cases ; 51% cures.

TABLE IV.—*Lognormality of Groups of Rectal Cancer Cases from St. Mark's Hospital, London*

| | Cases | Cancer deaths | Lognormality |
|---|---|---|---|
| A. Survey of 1932–1937 cases divided by stage | | | |
| Dukes' stage | | | |
| B | 84 | 21 | — |
| C | 138 | 90 | + |
| $C_1$ | 70 | 35 | + |
| $C_2$ | 65 | 53 | + |
| C—average grade | 64 | 35 | + |
| C—anaplastic | 67 | 52 | + |
| B. Survey of Dukes' B cases | | | |
| 5-Year period | | | |
| 1928–1932 | 71 | 28 | — |
| 1933–1937 | 84 | 21 | — |
| 1938–1942 | 103 | 33 | + |
| 1943–1947 | 173 | 47 | + |
| 1948–1952 | 221 | 65 | + |
| Location | | | |
| Low rectum | 221 | 64 | — |
| Mid rectum | 197 | 71 | — |
| Upper rectum | 234 | 59 | + |
| Histological grade | | | |
| Low | 148 | 39 | + |
| Average | 442 | 127 | — |
| Anaplastic | 62 | 28 | — |

became lognormal. The other factor was less apparent in large groupings since it was related to anaplasia and few anaplastic tumors were of Dukes' B stage.

When the anaplastic tumors were looked at alone, a biphasic pattern was seen. This was followed further, found to persist in other stages, and finally the subgroup of tumors responsible for the biphasic grouping of death times was identified. These were particularly lethal cancers, fully deserving of separate identification since they killed not only twice as fast but left only about half as many 5 year survivors as the cancers they had been mixed with.

Of about 100 subgroups studied in the rectal series, " unexplained " non-lognormality was found only once. Almost all of the groups that had non-lognormal distributions had them for a discoverable reason. Hence in general, the lognormal model seemed as applicable to treated rectal cancer cases as to the breast series and the other studied of patients treated for cure. This being the case, the instances in which the data did not fit the model were those most useful in case study.

### DISCUSSION

Probably the first question raised by this study is the validity of the visual approach to a decision. As authorities favoring the approach, one may cite Moore, *et al.* (1951) and Bliss (1937). The calculation of best fitting lines seems an attractive alternative at first glance but not so at second. Such calculation used a large amount of computer time ; the results rarely proved more than trivially better than the eye plots ; but most important, when there was a systematic deviation, the calculated line would tend to obscure it rather than point it up. No method has been suggested whereby one through computation could, with assurance, recognize systematic as opposed to non-systematic variations.

Accepting the present method then, the lognormal model often seemed applicable and a good description of groups of cancer cases with homogeneous treatment. To this degree the results of this survey support Boag's extrapolation. A simple way to try to achieve treatment uniformity is to consider only patients treated for cure. When no treatment is very successful, the model may also fit as it did the totality of lung cancer cases. When there are mixtures of effectively and ineffectively palliated cases, the model usually did not seem applicable, particularly with particular distributions of patients in these two groups. Insofar as the results may have in this way reflected a situation local to University College Hospital, they call for confirmation.

It would also be interesting to have longer-followed material for other types of cancer besides breast and rectal carcinoma since only with follow-up past the predicted 99% mortality time can one have real confidence in correspondence with the model. With earlier cut-off, a subsequent flurry of deaths could destroy what was till then an excellent fit. The lognormal model seems particularly sensitive to this situation—almost any assumption about cures still leads to a plot of death times that is linear in the first half of the data.

One example now exists that confirms the UCH observations for Hodgkin's disease. Neither there nor in the preliminary Memorial survey did the data fit the model. Hansen (1964) recently published an Australian series with survival curve data on which a lognormal study could be done. There again his group as a whole proved not to fit the model. However, when one removed the two small but very slowly lethal cases, those with "paragranulomas" and the newly-described "nodular sclerosing" variety, the remaining cases were in fact lognormally distributed. One thereby has added incentive to look closely at other groups that fail to fit the model to see if they too are composed of subgroups of importantly different survival.

Because of this property, a point taken for granted earlier in this presentation, probably should be spelled out to counteract misuse of this model. The model clearly applied to treated rectal and breast cancers only on the assumption that there were cures and only when the cured fraction of patients was separated off. It is true for most other cancers as well if cures appear to be achieved clinically. Stomach cancer cases from the UCH series were the most prominent exception I have encountered to this. If one feels that the no-cure situation applies, at least this should be specified so that the rationality of the assumption may be considered rather than just using say a probit curve to describe a total group of patients without adjustment.

The question of other models seems of more theoretical than practical importance. An exponential model has received the most attention (Berkson and Gage, 1952; Koldin, 1961) but its primary justification is simplicity. The Weibull model (Lieblein and Zelen, 1956) seems the most interesting alternative coming from industrial life testing which may or may not be analogous to survival with cancer. At the moment, none of the models follows from major established assumptions about cancer behavior. They must stand or fall on their merits, on how well they do their job. Their first job is to describe the series. The lognormal exponential and Weibull models all produced about the same figures for median survival time and cure rate in equivalent series tested at the time of writing.

The distribution of cases around this time also was not much different. In the first few months of a series, often no model fitted too well, though the exponential tended to be particularly poor as long noted.

We have yet too little data about truly late cancer behavior though this may well be an even more crucial region for choosing one model over another as far as adequacy of description goes.

Though we have some information on adequacy of description, we have as yet none on two two points that are of some importance in choosing a model : power in predicting final values of such figures as cure rates from incomplete data and power in making comparisons between groups of patients. In the first problem, a model is vital, in the second, important as an improvement over nonparametric comparisons. There is one place where the lognormal model has shown advantages yet to be claimed for the other models. Heterogeneous groups often are marked by systematic deviations from the model that are easy to see. With the other models the picture tends to be merely accentuation of expected deviations and recognition of significant cases has not seemed simple.

Another unexamined problem is the question of the most desirable initial point of time. Here, dates of diagnosis or first treatment were used. Time could also be computed from beginning of symptoms though this poses difficulties. It is a subjective date, known to be inaccurate. It may relate not to the cancer but to a precancerous state. Use of it means a loss of the cohort structure one has in taking all patients eligible in a given time interval, and it produces complications when there is a cured fraction (chance of cure often is correlated with symptom time). Still it might serve to bring the data closer to Bliss' original use in describing death times. He measured time from the administration of a toxin and so had a real time zero not possible here. The deaths still were lognormally distributed. Hopefully theory of cancer behavior will account for the agreement between his observations and the present ones.

### CONCLUSIONS

The degree of correspondence between lognormal models and actual death time data has been the subject of 3 complementary surveys involving about 250 partly overlapping groups of cancer patients. The requirement for the model to be applicable seems to be a continuity of patient material. Here treatment was the most obvious factor to be controlled within limits ; unless there was treatment for cure or no effective treatment at all, the model tended to fail. As an often good description of cancer death times, the model can serve to summarize data and simplify comparisons. In addition and most important for pathological studies, non-conformity, when unexpected, usually can be explained by patient or cancer differences that are thereby brought to light.

Most of this work was done while the author as recipient of an Alfred P. Sloan Foundation Award in Cancer Research, 1963, was with the Statistical Research Unit of the Medical Research Council, Dr. Richard Doll, Director. Much of the computation was performed on their Elliott 803 computer. Other work was done on another 803 at the Elliott Medical Automation Center, and on the CDC 160A of the Medical Physics Department, Memorial Hospital (USPHS Grant CA 06102). The author wishes to thank the many people who assisted him at these installations.

### REFERENCES

AITCHISON, J. AND BROWN, J. A. C.—(1963) 'The Lognormal Distribution'. Cambridge (Cambridge University Press).

BERG, J. W.—(1964) *Cancer*, **17**, 693.

BERKSON, J. AND GAGE, R. P.—(1952) *J. Am. statist. Ass.*, **47**, 501.

BLISS, C. I.—(1937) *Ann. appl. Biol.*, **24**, 815.

BOAG, J. W.—(1948) *Br. J. Radiol.*, **21**, 128.—(1948) *Ibid.*, **21**, 189.—(1949) *Jl R. statist. Soc.*, **11**, 15.—(1960) *Acta radiol.*, **54**, 289.

GADDUM, J. H.—(1945) *Nature Lond.*, **156**, 463.

HANSON, T. A. S.—(1964) *Cancer*, **17**, 1595.

KOLDIN, D.—(1961) *Cancer Res.*, **21**, 1103.

LIEBLEIN, J. AND ZELEN, M.—(1956) *J. Res. natn Bur. Stand.*, **57**, 273.

MOORE, F. J., CRAMER, F. B. AND KNOWLES, R. G.—(1951) 'Statistics for Medical Students'. New York (Blakiston).

ROBBINS, G. F. AND BERG, J. W.—(1964) *Cancer*, **17**, 1501.

TIVEY, H.—(1954) *Am. J. Roentg.*, **72**, 68.

University College Hospital, Cancer Follow-up Department—(1958) 'The Collected Statistics of Malignant Disease Seen at University College Hospital, London, During the Period 1946–1950'. Shrewsbury (Wilding and Son, Ltd.)